# Breast Reconstruction After Mastectomy Using a Deep Inferior Epigastric Perforator (DIEP) Flap: Clinical and Medico‐Legal Insights From a Four‐Year Study

**DOI:** 10.1002/hsr2.70499

**Published:** 2025-04-16

**Authors:** Demetris Savva, Giulio Nittari, Filippo Gibelli, Giovanna Ricci, Savvas Dalitis, Antonia Fotiou, Phanos Michael

**Affiliations:** ^1^ Department of Plastic Reconstructive and Aesthetic Surgery Nicosia General Hospital Nicosia Cyprus; ^2^ School of Pharmaceutical Sciences and Health Products University of Camerino Camerino Italy; ^3^ Section of Legal Medicine, School of Law University of Camerino Camerino Italy

**Keywords:** anatomy, global health, healthcare management, oncology

## Abstract

**Aims:**

Breast reconstruction after mastectomy is nowadays a gold standard in therapy of breast cancer patients. Free deep inferior epigastric perforator (DIEP) flap reconstruction is a favorable method when traditional implants fail or are not viable, especially after radiotherapy. The aim of this paper is to present the results of a case series study of 40 patients operated on with DIEP flap from January 2020 to October 2023, in Plastic, Reconstructive and Aesthetic Surgery Department in Nicosia General Hospital, Cyprus, complications and wound management, reoperation rates, as well as to examine these results from a medico‐legal perspective, to highlight the most significant medico‐legal implications of this demanding, surgical procedure.

**Method:**

Forty patients were included in this study with unilateral or bilateral free DIEP reconstruction, from January 2020 to October 2023, in Plastic, Reconstructive and Aesthetic Surgery Department in Nicosia General Hospital, Cyprus. Demographics, preoperative conditions, hospitalization days, complication rates, and reoperation rates were analyzed as well as satisfaction rates of patients were evaluated.

**Results:**

This original article highlighted a number of issues of strict medico‐legal interest, including the importance of informed consent in the case of demanding procedures for reconstructive and esthetic purposes, the assessment of standards of care in the evaluation of medical liability, and the existence of an obligation of means or results on the reconstructive surgeon's part.

**Conclusions:**

DIEP breast reconstruction after mastectomy is a challenging but safe and with well postoperative results operation that should be employed in cases traditional implant reconstruction fail or not feasible due to other parameters. Innovative and demanding reconstructive, esthetic surgery procedures are characterized by particularly significant aspects of medico‐legal interest, which deserve careful consideration by both the scientific community and patients involved.

## Summary

- Breast reconstruction after mastectomy is nowadays a gold standard in therapy of breast cancer patients.
- Deep inferior epigastric perforator (DIEP) breast reconstruction after mastectomy is a challenging but safe and with well postoperative results operation.
- Innovative and demanding reconstructive, esthetic surgery procedures are characterized by particularly significant aspects of medico‐legal interest.
- In breast reconstruction using DIEP flaps, informed consent is vital due to the procedure's innovative nature, microsurgical complexity, and variable outcomes.
- High surgical standards require skilled microsurgeons, and complication management must be proactive.

## 1 Introduction

Breast reconstruction after mastectomy is nowadays a standard part in the therapy of breast cancer patients, and no longer considered an optional complimentary secondary surgery in most countries at the moment. Throughout the years a lot of reconstruction modalities have been introduced to treat these patients; both autologous and alloplastic. The deep inferior epigastric perforator (DIEP) flap is considered a gold standard autologous option in the therapy of breast cancer patients [1, 2, 3].

DIEP flap reconstruction is by its nature a challenging, but safe operation with well postoperative results and high levels of satisfaction from both doctors and patients. However, a very well‐trained microsurgeon is necessary for a correct DIEP preparation and harvesting, as well as performing the crucial micro‐surgical anastomosis [4, 5]. This demanding but safe procedure requires continuous training of the microsurgeon to gain the experience and practical skills for performing these kinds of procedures with confidence and high rates of success [6].

## 2 Methods

For our study, 40 patients were included that underwent a unilateral or bilateral free DIEP reconstruction, from January 2020 to October 2023, in Plastic, Reconstructive and Aesthetic Surgery Department in Nicosia General Hospital, Cyprus.

The procedures performed in this study were in accordance with the ethical standards of the institution‐Plastic, Reconstructive and Aesthetic Surgery Department, Nicosia General Hospital‐ and with the 1964 Helsinki Declaration and its later amendments or comparable ethical standards. After explaining the purpose and the nature of the study, informed consent was obtained and signed from the subjects who accepted to participate. Anonymity and confidentiality of participants were ensured.

Patient selection plays a major factor in the success rate of these demanding surgeries. As a general rule, nonideal patients for undergoing a DIEP flap reconstruction are smokers, patients with previous surgeries in the donor area, hypertensive or diabetic patients, very thin patients, and patients over 65 years old. Once again these are only general rules, one must take into consideration the individual patient, his options, advantages, and disadvantages of each treatment option but also the patient compliance with demanding surgeries like this, that require more postsurgical attention that simpler reconstruction breast surgeries.

Another determining factor for the success of such surgeries is of course the quality of the microscope used as well as the experience of the microsurgeon himself in mastering this advanced microscope and achieve the necessary success in the surgery. In the case of this study, the microscope Zeiss®‐Tivato 700 was used. The specific microscope is used by our team due to various characteristics that excel it compared to others and help us provide successful results in demanding surgeries like this one. Again, the use of this high‐tech microscope always comes hand‐to‐hand also with the experience and training of the microsurgical team itself.

Tivato‐700 enables use of advanced surgical visualization modalities for both the doctor using the microscope as well as for the delivery image on the screen. Intraoperative fluorescent modalities, allows us to check live and fast the patency of the anastomosis. 4K visualization on the external screen and for broadcasting in brilliant quality and detail allows the surgery to be followed easily by attending doctors as well as for educational purposes. And lastly, being an all‐digital microscope enables us easy patient recording of data as well as video or photographic documentation easily registered in the platform and access from any device need.

All patients were treated by the same attending plastic surgeon and his team and followed the same postsurgical care for the purpose of this study.

## 3 Results

Demographics, preoperative conditions, hospitalization days, complication rates, and reoperation rates were analyzed as well as satisfaction rates of patients were analyzed.

**Figure 1:**
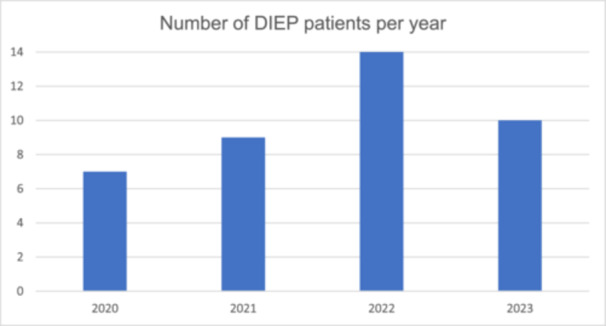
Number of deep inferior epigastric perforator (DIEP) patients per year.

In 2020, seven patients were treated with DIEP flap reconstruction, one of them bilaterally. In 2021, nine patients with one bilateral surgery. In 2022, 14 patients with three bilateral, and lastly, in 2023 10 patients with two bilateral reconstructions (Figure 1).

**Figure 2:**
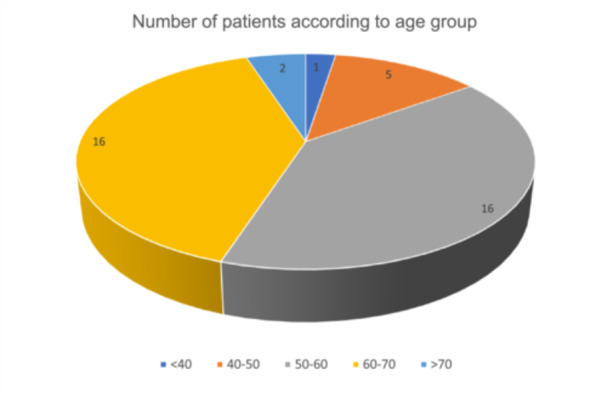
Number of patients according to age group.

Patients were then grouped into age groups for the purpose of our study as shown in Figure 2. The majority of our patients were between ages 50 and 70. Two patients, one 71 and one 72‐year‐old, were operated on successfully despite their age. These candidates were chosen keeping into consideration their clear medical anamnesis, their good health condition as well as their compliance postsurgical.

**Figure 3:**
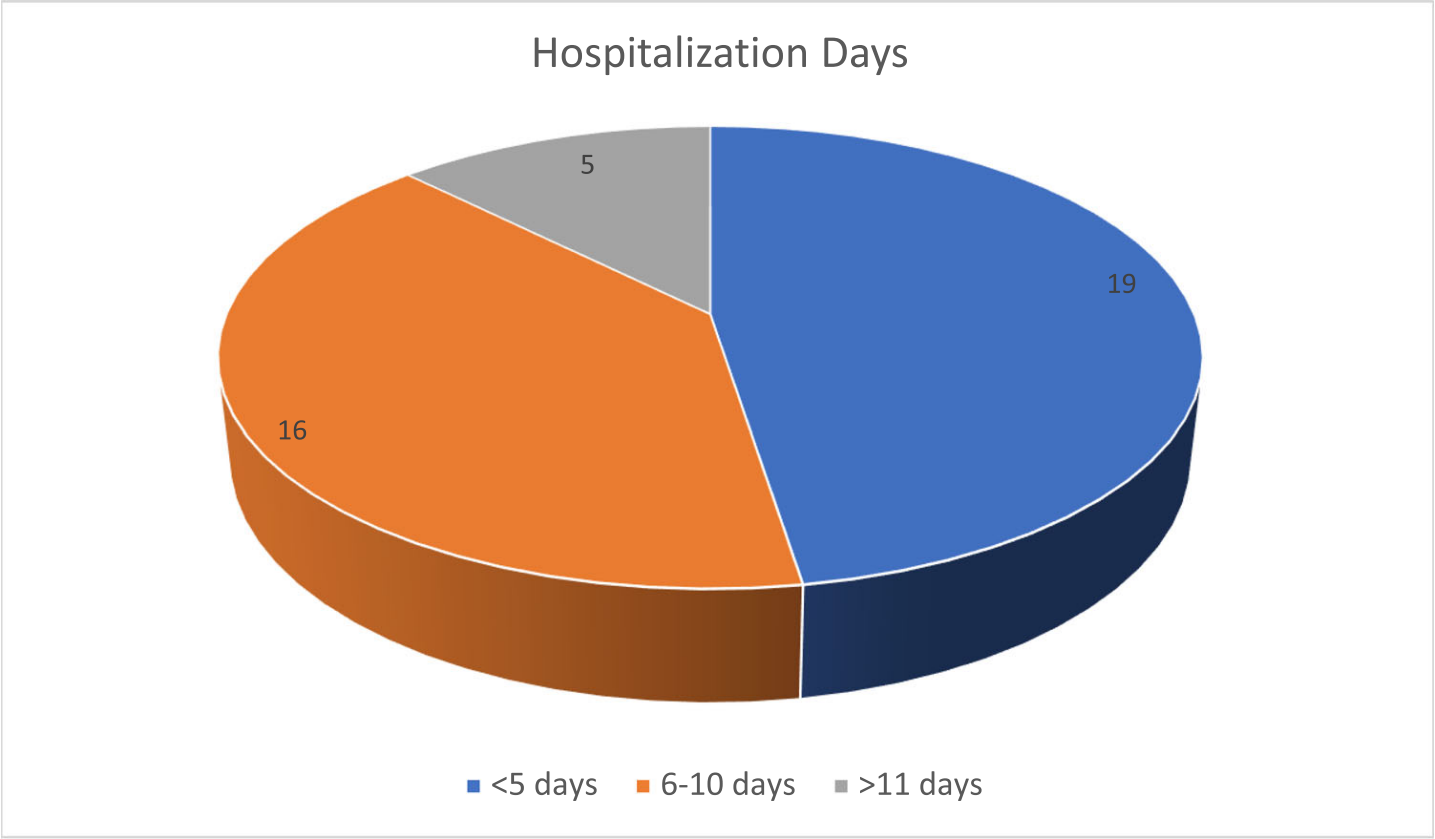
Hospitalization days.

Patients were admitted the night before surgery at the ward, having done all the necessary preoperative imaging and laboratory examinations. After operation they stayed at the ward for around 5–7 days for flap monitoring as well as until they were physically able to stand and tend themselves. Hospitalization days of our patients are shown in Figure 3.

A standardized chemoprophylaxis protocol of one dose in‐operatively and three doses postoperatively of second generation cephalosporin were used in all patients. Routine Monitoring of the flap was done with doppler sonography every 1 h for the first 24 h, and then every 3–4 h for the next 48 h. Physical examination was also used to assess the viability of the DIEP flaps, more specifically warmth and color, capillary refill and turgor. Pinprick test was used in ambiguous cases to verify the viability of the flap and to distinguish between epidermal necrolysis and necrosis of the periphery of the flap [7, 8, 9, 10]. Patients were encouraged to stand within 24 h after the surgery with the use of abdominal binder and nursing assistance.

**Figure 4:**
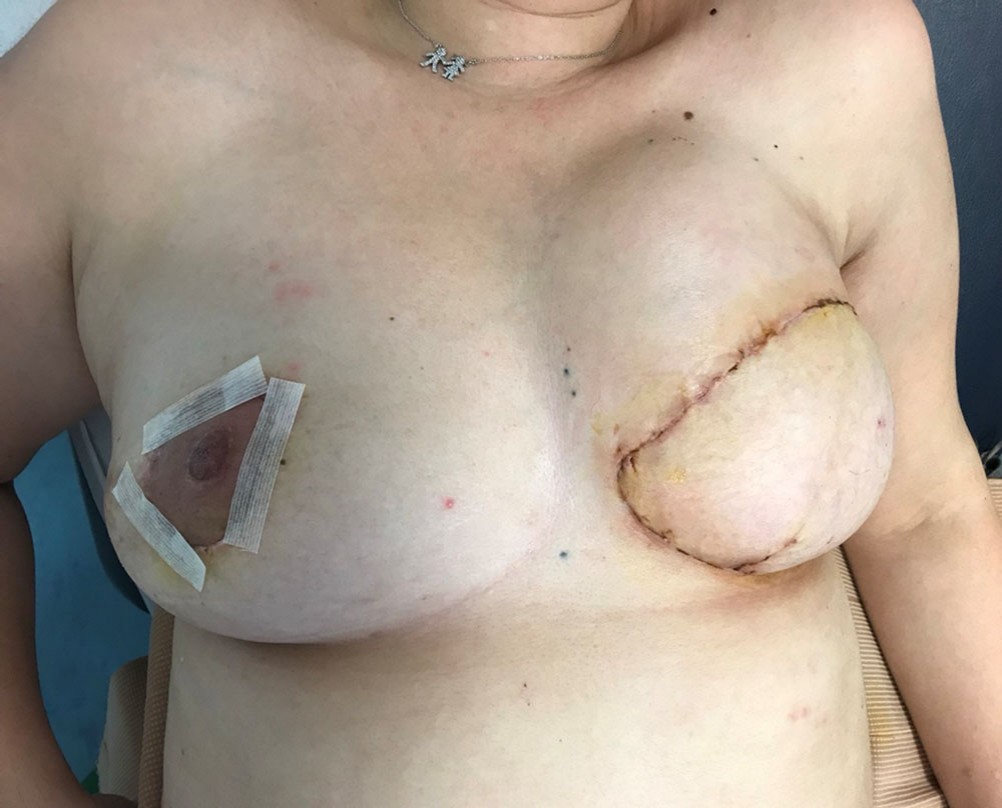
One day after postop.

**Figure 5:**
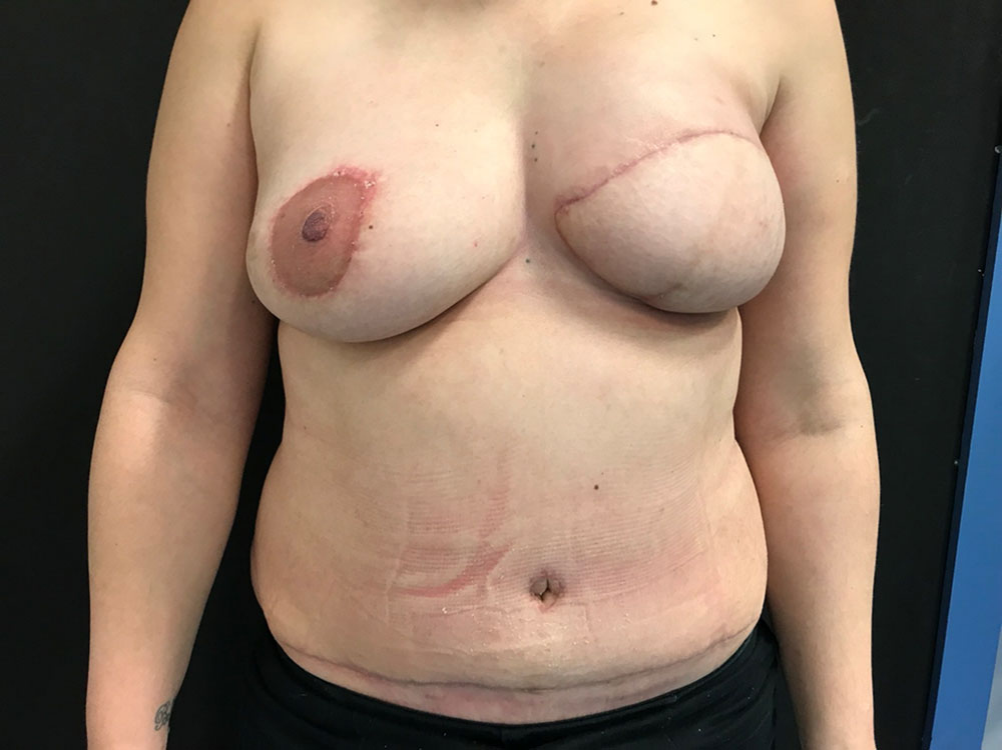
Two months postop.

Figures 4 and 5 show a 45‐year‐old patient that underwent right mastopexy and left DIEP reconstruction.

**Figure 6:**
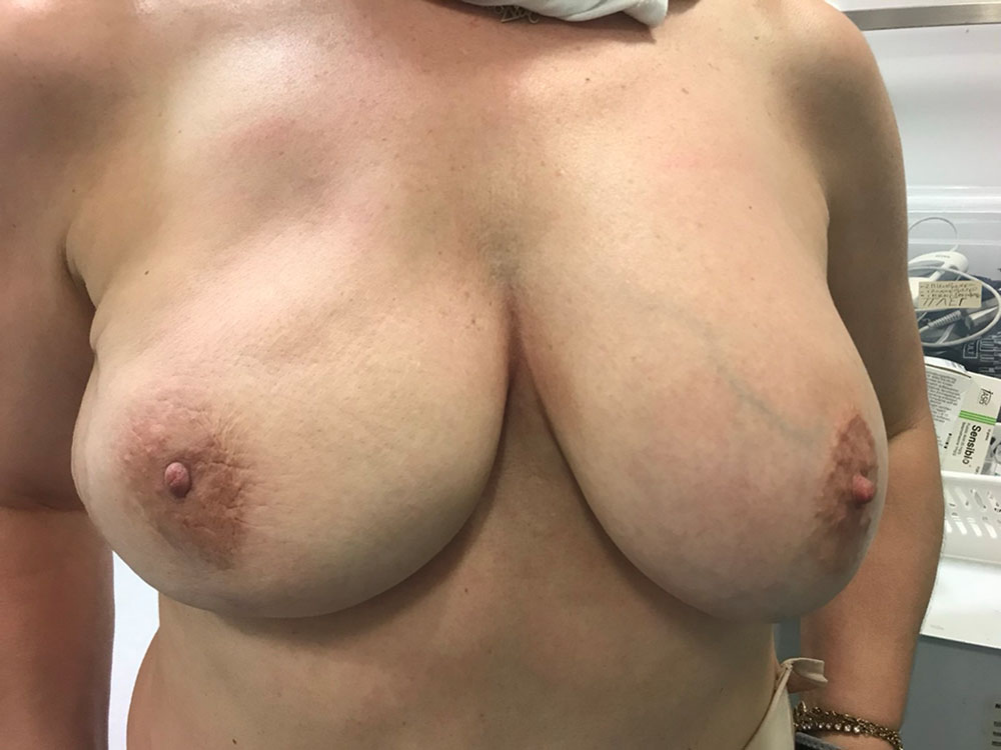
Three month post op.

**Figure 7:**
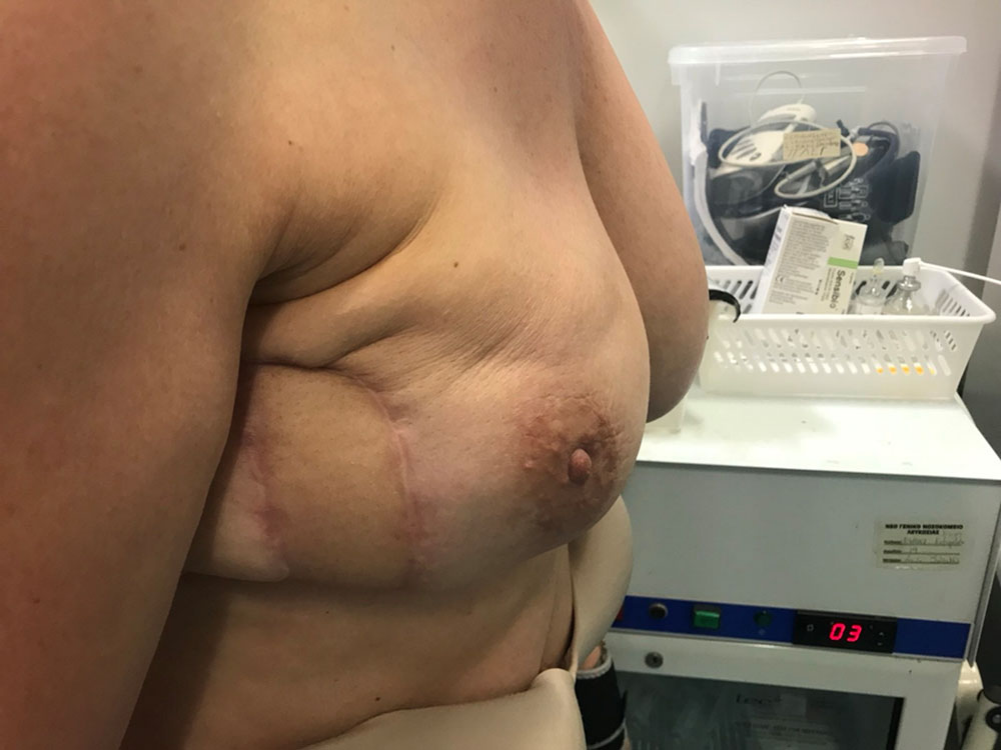
Three month post op‐lateral view.

Figures 6 and 7 show a 52‐year‐old patient 3‐month post‐op special case that was treated with a DIEP flap due to contractures on lateral side and nipple distortion following breast lumpectomy and axillary lymph node dissection following a positive sentinel lymph node biopsy. Patient was suggested a mastopexy with or without implant but with a possible mammoplasty on second breast for balancing. She decided not to include her contralateral breast in the surgery and a DIEP reconstruction was finally performed on the right breast.

Postsurgical complications were minimal; five patients developed a seroma at the donor site, three patients developed an infection of surgical wound site that resolved with some additional days of antibiotic treatment and lastly only one DIEP flap was lost despite all salvaging attempts that was eventually replaced with a latissimus dorsi (LD) flap [11].

**Figure 8:**
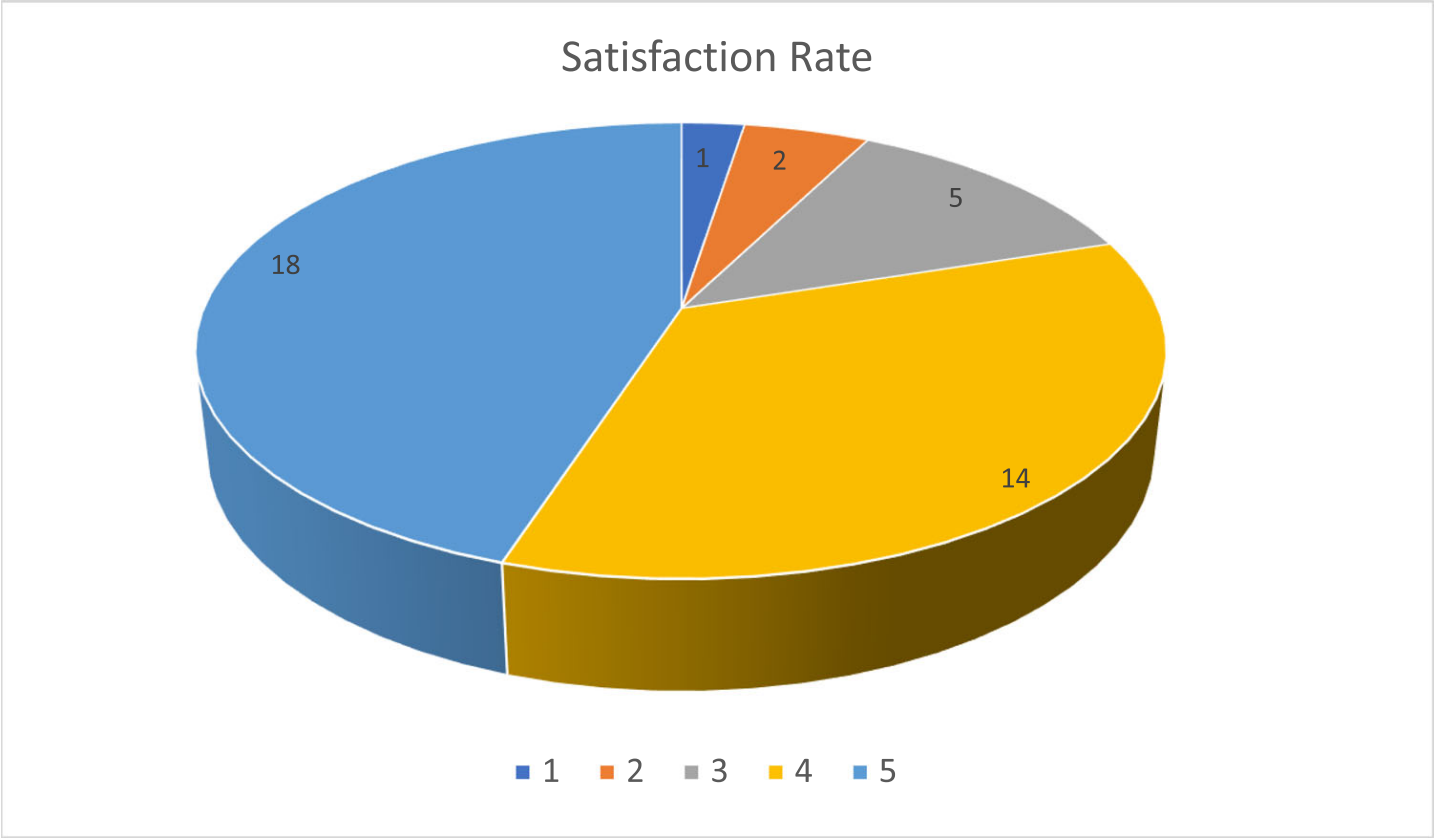
Satisfaction rate.

Upon leaving the hospital, patients were monitored in the outpatient clinic of our department at regular intervals according to need and at 2‐month follow‐up they were asked to evaluate their experience of the surgery itself, on their doctors and the hospital facility using the Likert scale (1: not satisfied at all, 2: unsatisfied, 3: neutral, 4: satisfied, and 5: very satisfied). More than half of the patients were fully satisfied with the whole process as it can be seen analytically on Figure 8.

## 4 Discussion and Medico‐Legal Insights

In the context of breast reconstruction with DIEP flap, several medico‐legal and medico‐judicial implications need to be highlighted.

First there is, of course, the issue of informed consent. With regard to breast DIEP reconstruction technique under consideration, the patient must always be aware of undergoing such a demanding procedure, technically complex and based on a big scale on the experience of the microsurgeon, entailing a series of advantages but also implying a series of quite peculiar complications for which a patient undergoing a normal breast reconstruction with or without implants will generally not have to take into consideration. On the one hand, the use of autologous tissue instead of alloplastic tissue provides a series of advantages, like a more natural result, using patient's tissue, and so forth, on the other hand we must also consider that the final result will in some ways be more “unpredictable” than with a traditional implant technique. In fact, since it is an adipocutaneous flap, it will behave like natural tissue, so the patient should be aware that breast reconstructed with a DIEP flap will undergo esthetic variations exactly like natural breasts (unlike breasts undergoing breast augmentation using a silicone implant). The patient should also be aware of the fact that the technique will result in a horizontal scar on the lower abdomen, as well as of the fact that a necrosis of the flap could occur giving the surgeon the possibility to harvest the contralateral side, resulting in a long scar similar to esthetic abdominoplasty (although this is a rare complication), or even need to harvest a flap from another area of the body (i.e., latissimus dorsi flap).

Therefore, patient careful information is particularly important, explaining thoroughly the nature of the operation, giving the fact that it is a microsurgical operation, with all its advantages and disadvantages following such an operational microsurgical technique.

It should also be recalled that in general terms, in any plastic surgery operation for esthetic purposes, the information activity requires a particularly high degree of rigor, precisely because these are medical acts (and as such entail health risks) carried out in the face of a nonurgent need for intervention (unlike in the case of operations performed to treat pathological conditions), despite the fact that such surgeries after breast tumor resection are not considered 100% an esthetic procedure nowadays, but part of the reconstruction postmastectomy.

A second aspect of particular interest from a medico‐legal point of view is that of surgical standards and the competence of the surgeon itself. The standard of care in DIEP flap reconstruction requires a highly qualified microsurgeon and the use of advanced technology, such as the Zeiss®‐Tivato 700 microscope. The competence and experience of the surgeon are of course crucial for the success of the operation. In general terms, in the event of a failure attributable to professional liability (i.e., not to unforeseeable or preventable complications), the surgeon is liable if he or she failed to observe the basic prudent and diligent surgical practices of the average professional.

It is evident that when we are talking about particularly complex operations or involving high microsurgical standards, determining the degree of deviation from average professional conduct is more complex. In the event of a preventable adverse outcome, determining the extent of the deviation between the surgeon's conduct and the conduct that should have been implemented is of fundamental importance, not only to assess whether liability profiles can be configured, but also to assess the possible existence of liability profiles for “gross negligence,” an aspect that has important implications in terms of administrative liability in various legal systems (such as the Cypriot and Italian system). Moreover, when surgical standards are particularly high, the excuse of “special difficulty” could be invoked.

A third aspect of medico‐legal interest that deserves to be examined in depth is that relating to the management of complications. Complications are inherent in any surgical procedure and DIEP flap reconstruction is no exception. Seroma formation, infection and flap failure represent some of the potential postoperative complications that need to be managed proactively. The legal implications of complication management are significant, as failure to diagnose, treat in a timely manner, or follow standard protocols can clearly lead to complications and therefore possible liability charges. It is therefore crucial to have clear, evidence‐based guidelines for the management of complications and to ensure that each intervention is thoroughly documented in the patient files.

The long‐standing issue of the distinction between obligations of means and of results in cosmetic surgery also deserves consideration. Obligations of “means” imply that the debtor (the doctor) must competently perform a given service regardless of whether the specific objective pursued by the creditor (the patient) is achieved, while obligations of “result” imply that the debtor must achieve the specific objective pursued by the creditor. If, in medical activities in general, the orientation that an obligation of means only is incumbent on the doctor is fairly well established, the issue is more complex with respect to cosmetic surgery, which, according to numerous interpretations, should provide for an obligation of result because of its particular purpose. It should be briefly recalled how in reality this orientation has mostly been superseded, configuring an obligation of means alone also on the part of the cosmetic surgeon. However, in innovative cosmetic/reconstructive surgery, if a technique is advertised as being able to guarantee certain results, the surgeon could be seen as obliged to achieve those results, thus bringing his liability closer to an obligation of result. In summary, therefore, while in traditional cosmetic surgery there is predominantly an obligation of means, the advent of innovative techniques could shift the balance towards obligations of results, requiring practitioners to be more cautious in communicating the potential and limitations of the proposed operations.

Another aspect that deserves consideration is that of patient selection and preoperative assessment. As mentioned, the selection of suitable candidates for DIEP flap reconstruction is a complex and not always straightforward process. The exclusion of patients with risk factors such as smoking, previous surgery or comorbidities must be balanced against the patient's right to benefit from such a treatment with important advantages over traditional techniques. Careful preoperative assessment and clear documentation of the reasons behind patient selection are essential to defend against any claims of discrimination or unfair denial of treatment.

Finally, it is considered useful to propose a brief reflection on patient satisfaction. Patient satisfaction is increasingly recognized as a measure of the quality of medical care. The use of satisfaction metrics, such as the Likert scale, provides valuable feedback for quality improvement initiatives. However, these metrics can also be indicative of the standard of care provided and can be examined in legal contexts. Ensuring high levels of patient satisfaction can serve as a protective factor against claims for negligence or dissatisfaction with medical services.

Given the time period of our study and the Covid pandemic, the number of patients can be considered a reduced sample, but our clinic is the only public clinic in the country providing this kind of surgery in a mass scale and the operations were not limited by the pandemic. Therefore, the pool sample is representative according to the country's population and provide a reliable insight to the kind of surgery.

## 5 Conclusion

DIEP breast reconstruction after mastectomy is a challenging operation but as we have concluded in our study that is a safe and with well postoperative results operation, with minor implications on wound management and with low percentage of wound infection, with a well compliant patient.

A trained and skillful microsurgeon and a good team, patient selection and compliance are the fundamental key elements for success. DIEP reconstruction after mastectomy should be considered more often as a treatment option despite its challenges and should be employed in cases traditional implant reconstruction fail or not feasible due to other parameters, therefore skipping some steps in the traditional reconstructive ladder and “using” the elevator [12].

In breast reconstruction using DIEP flaps, informed consent is vital due to the procedure's innovative nature, microsurgical complexity, and variable outcomes. High surgical standards require skilled microsurgeons, and complication management must be proactive. Liability shifts toward obligations of results with innovative techniques, necessitating careful communication about potential and limitations. Proper patient selection and thorough documentation are essential, and patient satisfaction metrics are important for both quality care and legal protection.

## Author Contributions

**Demetris Savva:** data curation, formal analysis, methodology, writing–review and editing. **Giulio Nittari:** conceptualization, data curation, investigation, project administration, writing–original draft, writing–review and editing. **Filippo Gibelli:** data curation, validation, visualization, writing–review and editing. **Giovanna Ricci:** investigation, supervision, visualization. **Savvas Dalitis:** data curation, formal analysis, methodology, writing–review and editing. **Antonia Fotiou:** data curation, investigation, validation. **Phanos Michael:** conceptualization, supervision, validation, writing–original draft, writing–review and editing.

## Ethics Statement

The procedures performed in this study were in accordance with the ethical standards of the institution‐Plastic, Reconstructive and Aesthetic Surgery Department, Nicosia General Hospital‐ and with the 1964 Helsinki Declaration and its later amendments or comparable ethical standards.

## Consent

After explaining the purpose and the nature of the study, informed consent was obtained and signed from the subjects who accepted to participate. Anonymity and confidentiality of participants were ensured by the authors.

## Conflicts of Interest

The authors declare no conflicts of interest.

## Transparency Statement

The lead author Giulio Nittari affirms that this manuscript is an honest, accurate, and transparent account of the study being reported; that no important aspects of the study have been omitted; and that any discrepancies from the study as planned (and, if relevant, registered) have been explained.

## Data Availability

The data that support the findings of this study are available from the corresponding author upon reasonable request.
